# Supplementary material for: A diminutive new basilosaurid whale reveals the trajectory of the cetacean life histories during the Eocene
Source: Commun Biol. 2023 Aug 10;6:707. doi: 10.1038/s42003-023-04986-w (PMC10415296; doi:10.1038/s42003-023-04986-w)
Supplement: Supplementary file 6 — Supplementary Data 3 [file 42003_2023_4986_MOESM6_ESM.zip › Supplementary Data 3/Supplementary Data 1_BTD_ASR/trait_0177_tree.plot.pdf]

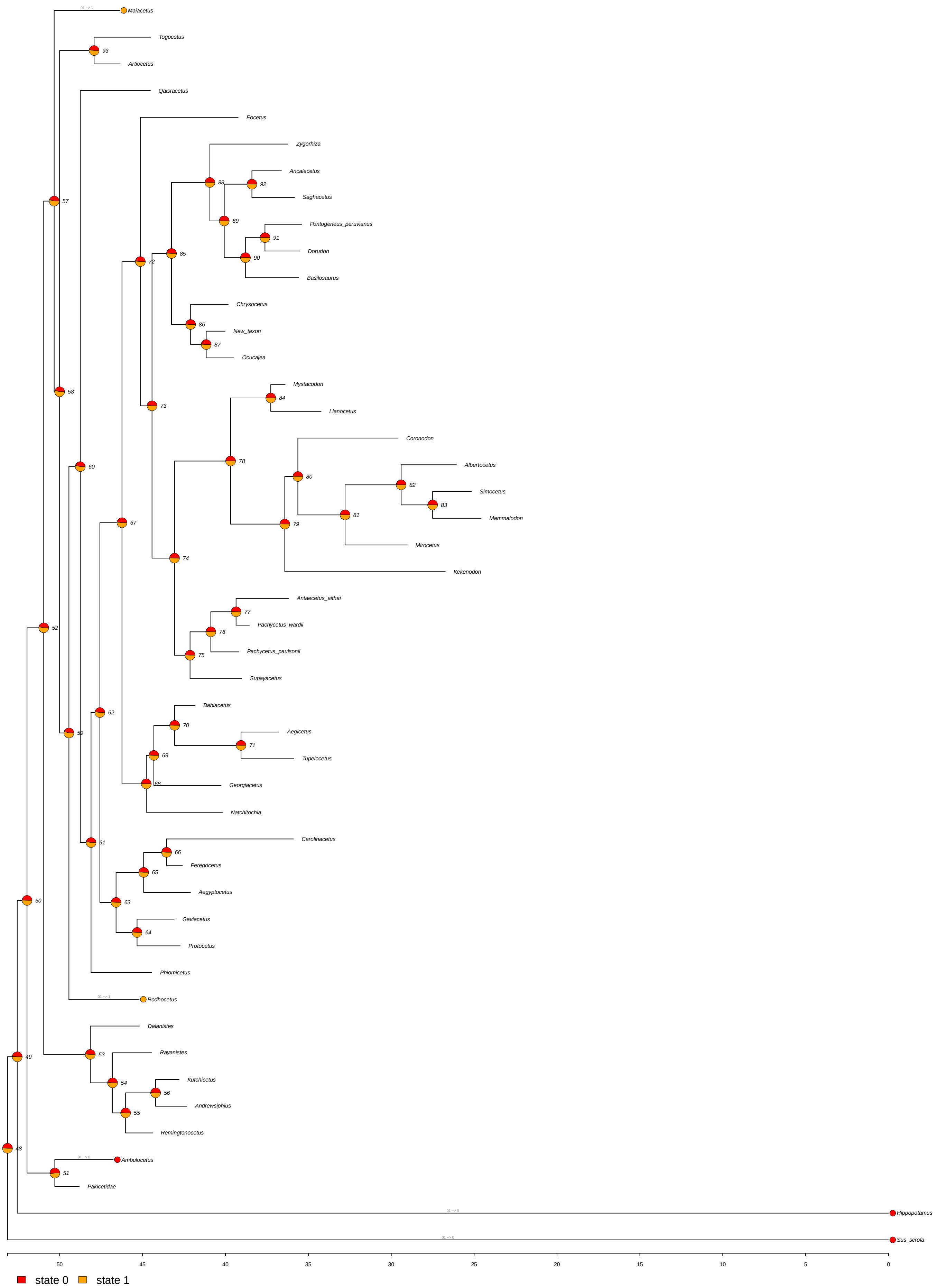
Maiacetus
Togocetus
Artiocetus
Qaisracetus
Eocetus
Zygorhiza
Ancalecetus
Saghacetus
Pontogeneus_peruvianus
Dorudon
Basilosaurus
Chrysocetus
New_taxon
Ocucajea
Mystacodon
Llanocetus
Coronodon
Albertocetus
Simocetus
Mammalodon
Mirocetus
Kekenodon
Antaecetus_aithai
Pachycetus_wardii
Pachycetus_paulsonii
Supayacetus
Babiacetus
Aegicetus
Tupelocetus
Georgiacetus
Natchitochia
Carolinacetus
Peregocetus
Aegyptocetus
Gaviacetus
Protocetus
Phiomicetus
Rodhocetus
Dalanistes
Rayanistes
Kutchicetus
Andrewsiphius
Remingtonocetus
Ambulocetus
Pakicetidae
Hippopotamus
Sus_scrofa
50 45 40 35 30 25 20 15 10 5 0
state 0
state 1
